# Language disturbances in schizophrenia: the relation with antipsychotic medication

**DOI:** 10.1038/s41537-020-00114-3

**Published:** 2020-09-07

**Authors:** J. N. de Boer, A. E. Voppel, S. G. Brederoo, F. N. K. Wijnen, I. E. C. Sommer

**Affiliations:** 1 Department of Psychiatry, University Medical Center Utrecht, Utrecht University & Brain Center Rudolf Magnus, Utrecht, the Netherlands; 2 University of Groningen, University Medical Center Groningen, Department of Neuroscience and Department of Psychiatry, Groningen, the Netherlands; 3 Utrecht Institute of Linguistics OTS, Utrecht University, Utrecht, the Netherlands

**Keywords:** Schizophrenia, Biomarkers

## Abstract

Language disturbances are key aberrations in schizophrenia. Little is known about the influence of antipsychotic medication on these symptoms. Using computational language methods, this study evaluated the impact of high versus low dopamine D_2_ receptor (D2R) occupancy antipsychotics on language disturbances in 41 patients with schizophrenia, relative to 40 healthy controls. Patients with high versus low D2R occupancy antipsychotics differed by total number of words and type-token ratio, suggesting medication effects. Both patient groups differed from the healthy controls on percentage of time speaking and clauses per utterance, suggesting illness effects. Overall, more severe negative language disturbances (i.e. slower articulation rate, increased pausing, and shorter utterances) were seen in the patients that used high D2R occupancy antipsychotics, while less prominent disturbances were seen in low D2R occupancy patients. Language analyses successfully predicted drug type (sensitivity = 80.0%, specificity = 76.5%). Several language disturbances were more related to drug type and dose, than to other psychotic symptoms, suggesting that language disturbances may be aggravated by high D2R antipsychotics. This negative impact of high D2R occupancy drugs may have clinical implications, as impaired language production predicts functional outcome and degrades the quality of life.

## Introduction

Disorder language is a hallmark feature of schizophrenia. Varying degrees of aberrant language can be observed in up to 80% of patients with schizophrenia^1^, and have been shown to comprise a broad variety of abnormalities in semantics, syntax, and phonology^2–5^. Most common among these are poverty of speech (alogia), increased pausing, reduced variation in intonation (monotone speech), and disturbances in the (discursive) coherence, such as derailment and tangentiality^6–8^. Since language is of primary importance for social relations and daily interactions^9^, it is a worrisome observation that language is affected in this patient group, as patients with schizophrenia are known to experience difficulties in maintaining trustworthy relations with others^9^ and are at an increased risk of social isolation^10^. Language disturbances in schizophrenia may negatively impact social relations in several ways. For example, reduced speech rate is known to negatively influence judgments of the speaker by others. Slower speakers are considered less truthful, less fluent, and less persuasive^11,12^. Disturbances in spontaneous speech can therefore have a negative impact on a broad range of life experiences^13^. Moreover, abnormalities of language in schizophrenia are predictive of functional outcome^14,15^, have a negative impact on both objective and subjective quality of life^16^, and thus greatly impact rehabilitation.

Antipsychotics are considered the first choice of treatment for schizophrenia, with positive effects reported on hallucinations, delusions, and disorganization^17–19^. However, little is known about the remedying effects of antipsychotic medication on language disturbances. Importantly, there is reason to assume that antipsychotic drugs may contribute to some of these language perturbations^20,21^. Specifically, antipsychotic drugs’ effects on dopamine receptors can be hypothesized to impair language, in several ways.

Firstly, negative language symptoms with a cognitive basis, such as poverty of speech and incoherence, are likely to be affected by antipsychotic medication, since antipsychotic drugs potentially increase these symptoms by blocking dopamine receptors in prefrontal brain areas that are thought to be hypodopaminergic in patients with a psychosis^22–24^.

Secondly, binding to the striatal dopamine receptor is known to cause extrapyramidal side effects (EPS), such as tremor, bradykinesia and rigidity^25,26^. Although EPS are classically known as limb movement disturbances, they also affect the motor components of spoken language production, i.e., the programming and execution of articulatory movements^27^. Indeed, drug-induced EPS, or severe Parkinsonism, is characterized by slow, hesitant and soft speech, indicating that medication affects planning and controlling of articulatory movements^27^.

While all antipsychotic drugs block the dopamine D_2_ receptor (D2R), some do so quite extensively and others more subtly. They can be classified based on their mechanism of action. For example, clozapine and quetiapine bind more loosely to the D2R than dopamine itself (henceforth low D2R occupancy drugs)^28^. By contrast, typical antipsychotics such as haloperidol and risperidone are “strong” D2R antagonists (henceforth high D2R occupancy drugs), as they bind more tightly to the receptor than dopamine. It can, therefore, be expected that not all types of antipsychotic medication will have similar effects on language production. Rather, the extent to which the medication binds to dopamine receptors may play a vital role in this.

Language production involves at least three processing systems: the conceptualizer, the formulator and the articulator^29^. Conceptualizing involves the organizing of ideas and intentions into a preverbal message. The formulator translates this preverbal message into a linguistic structure with its corresponding meaning and form. Finally, articulation involves the programming and execution of a predetermined phonetic plan by the muscles of the articulatory tract. The processing systems involved in language production can therefore also be categorized as being either primarily cognitive (conceptualizer and formulator) or motoric (articulator) in nature. Language production is thus a shared motoric and cognitive process, and is therefore likely to be affected by dopamine blockage in both striatal as well as prefrontal brain areas.

Previous studies assessing language and speech in schizophrenia revealed that at the level of speech delivery, proportion of spoken time and speech rate are decreased, and (clause initial) pauses are increased^30–32^. As regards language structure and content, research indicates that syntactic complexity is decreased^6^, which is reflected in short sentences with reduced embedding and limited lexical diversity. Furthermore, schizophrenia patients suffer from word-finding issues (mostly related to content words such as nouns or verbs), resulting in longer pauses and disfluencies^6^.

Summarizing, language disturbances are a core symptom of schizophrenia, which greatly impacts social and functional outcomes and quality of life. Little is known about the impact of antipsychotic medication on language in patients with schizophrenia. As EPS can negatively affect the articulatory system (i.e., programming and execution of articulatory movements), and brain areas implicated in the cognitive components of language production (conceptualizing and formulating) are known to be negatively affected by antipsychotic drugs that block dopamine receptors, such drugs may have a relatively negative impact on spoken language in schizophrenia patients. From the above follows the hypothesis that language will be more severely disturbed (e.g. increased pauses and slower speech rate) in patients with schizophrenia that use high D2R occupancy medication than in those using low D2R occupancy medication. In the present study, we set out to test this hypothesis by comparing spoken language samples of schizophrenia patients on language variables that are known to be disturbed in schizophrenia^6,30,31,33^ (see Table 1 for an overview). Patients were divided into two categories based on dopamine binding profiles, namely patients with low D2R occupancy drugs (i.e. quetiapine, paliperidone, olanzapine, and clozapine) or high D2R occupancy drugs (i.e. aripiprazole, risperidone, flupentixol, amisulpride, and haloperidol). We additionally analyzed language produced by a healthy control group for comparison and explored the relation with psychotic symptom severity.

**Table 1:** Description of language variables.

Variable	Definition/calculation	Measures
Total number of words	Total words produced in the interview	Indicative of poverty of speech.
Articulation rate	Syllables/phonation time	(Motor) speed in speech production.
Average pause duration	Total time the participant was pausing in seconds/number of pauses	Pauses often reflect formulating or planning language and might therefore reflect processing speed.
Average turn duration	Average duration of a speaking turn in seconds	Average length of an answer, before another question is necessary.
Percentage of time speaking	Time participant speaking/time interviewer speaking*100	Might reflect spontaneity in speech or willingness to speak.
Mean length of utterance (MLU)	Mean length of utterance in morphemes	Sentence complexity. Greater length indicates more complex sentences.
Type-token ratio (TTR)	# types/# tokens	Lexical diversity. Types: the number of different words used in the sample. Tokens: all words in the sample. This number goes from 0.001 to 1.0. Low values indicate a lot of repetition, high values means each word in the sample was different. High TTR indicates fewer syntactical structures.
Clauses per utterance	Average number of clauses per utterances	Grammatical complexity. More clauses per utterance indicate more syntactical complex sentences.
Noun verb ratio	# nouns/# verbs	Number of nouns per verbs. Might reflect specific difficulty with either nouns or verbs.
Open–closed ratio	# open class words/# closed class words	Content words versus function words. Open class: content words. Word class accepts new members easily. Closed class: function words. Word class does not easily accept new members. Might reflect specific difficulty with either content or function words.
Disfluencies	# of disfluencies/# all words	Difficulties formulating sentences. All forms of disfluencies, including filled pauses and retracing as a percentage of all words.
Pause to word ratio	# pauses/# all words	Indication of processing speed. Measures how many pauses are needed to formulate one word.

## Results

### Demographics

Clinical and demographic data are shown in Table 2. The groups did not differ with regard to sex and age. Patients had received less education than healthy controls, and there was no difference in parental education level between groups. Symptom severity as measured by the Positive And Negative Syndrome Scale (PANSS) as well as drug dose as measured in chlorpromazine equivalent dose did not differ between patients with high or low D2R occupancy medication.

**Table 2:** Demographic characteristics of patients with schizophrenia with high 2DR occupancy medication, low D2R occupancy medication, and healthy controls.

	High D2R	Low D2R	HC	Test statistic	*p*-value
*n*	23	18	40		
Sex, male: female, *n*	17:6	14:4	36:4	χ^2^ = 3.038	0.219
Age, mean (SD)	28.5 (9.04)	28.3 (9.39)	31.7 (11.71)	F(2,78) = 1.002	0.372
Years of education, mean (SD)	13.2 (2.63)	12.2 (2.94)	14.8 (2.20)	F(2,77) = 7.446	0.001
Parental years of education, mean (SD)	12.7 (2.93)	12.3 (2.85)	12.8 (3.10)	F(2,68) = 0.128	0.880
Self-reported language fluency, *n*					
Fluent in Dutch only	13	15	19	χ^2^ = 7.357	0.118
Fluent in two languages	9	3	17		
Fluent in three language	1	0	4		
Duration of illness years, mean (SD)	4.5 (5.55)	5.1 (7.49)		MW = 189	0.871
Total PANSS, mean (SD)	52.0 (11.98)	52.1 (8.57)		F(1,39) = 0.002	0.963
PANSS positive	10.7 (4.72)	11.2 (2.98)		F(1,39) = 0.136	0.714
PANSS negative	14.0 (5.12)	14.9 (4.96)		F(1,39) = 0.313	0.579
PANSS general	27.3 (6.51)	26.1 (4.95)		F(1,39) = 0.424	0.519
Psychotic disorder*, n*				χ^2^ = 4.244	0.236
Schizophrenia	8	5			
Schizoaffective disorder	4	3			
Schizophreniform disorder	0	3			
Psychosis NOS	12	7			
Chlorpromazine equivalent (mg), mean (SD)	347.8 (217.87)	518.6 (302.9)		MW = 107.5	0.056
Antipsychotic medication, *n*					
Amisulpride	2				
Aripiprazole	15				
Flupentixol	1				
Haloperidol	3				
Risperidone	2				
Clozapine		5			
Olanzapine		5			
Paliperidone		4			
Quetiapine		4			

*SD* standard deviation, *n* sample size, *High D2R* schizophrenia patients with high dopamine D2 receptor occupancy medication, *Low D2R* schizophrenia patients with low dopamine D2 receptor occupancy medication, *HC* healthy controls, *PANSS* positive and negative syndrome scale, *NOS* not otherwise specified, *MW* Mann–Whitney U.

### Language variables between the groups

**Fig. 1:**
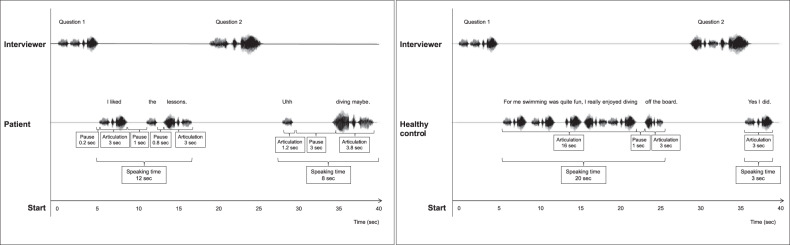
Illustration of the interview and language measures. The speech waves (oscillograms) are for illustrative purposes only and do not reflect the actual recordings of these sentences.

For an overview of the language variables, see Fig. 1 and Table 1. Schizophrenia patients with high versus low D2R occupancy medication and healthy controls were compared on language variables using a MANCOVA.

To correct for the influence of age, sex, and education level on normal variation in language, these variables were entered as covariates in the model. The MANCOVA revealed a main effect of group status on language (Pillai’s trace = 0.526, F (2, 80) = 1.843, *p* = 0.016, partial η^2^ = 0.263). Furthermore, a main negative effect was found for age (Pillai’s trace = 0.328, F (1, 80) = 2.477, *p* = 0.010, partial η^2^ = 0.328) and male sex (Pillai’s trace = 0.395, F(1, 80) = 3.315, *p* = 0.001, partial η^2^ = 0.395), but not for education level. No interaction effects were found between the independent variables. Post-hoc tests revealed that patients that use high D2R occupancy drugs differ from low D2R occupancy patients on a number of language variables, including total number of words and type-token ratio (TTR, i.e. a measure for lexical diversity), as well as from healthy controls on several language variables, including articulation rate, TTR, mean length of utterance (MLU). However, low D2R occupancy patients do not differ from healthy controls on most aspects of language (see Table 3).

**Table 3:** Language characteristics of patients with schizophrenia with high 2DR occupancy medication, low D2R occupancy medication, and healthy controls.

Language variables	(A) High D2R *n* = 23	(B) Low D2R *n* = 18	(C) HC *n* = 40			Post-hoc analyses^a^		
	Mean ± SE	Mean ± SE	Mean ± SE	F-statistic	*p*-value	A vs B	B vs C	A vs C
Total number of words	1043.0 ± 113.60	1485.6 ± 140.56	1768.9 ± 101.18	11.544	<0.0001**	0.025*	–	<0.0001**
Articulation rate	5.5 ± 0.20	5.9 ± 0.25	6.2 ± 0.18	3.156	0.049*	–	–	0.017*
Pause duration (s)	1.03 ± 0.043	0.90 ± 0.053	0.86 ± 0.038	2.850	0.064	–	–	0.006**
Speaking turn duration	8.7 ± 1.54	7.2 ± 1.90	10.8 ± 1.37	0.684	0.508	–	–	–
Percentage of time speaking	69.2 ± 1.98	70.0 ± 2.45	77.3 ± 1.76	4.999	0.009**	–	0.029*	0.004**
MLU	13.4 ± 1.37	16.8 ± 1.69	19.1 ± 1.22	6.539	0.002**	–	–	0.003**
TTR	0.20 ± 0.008	0.17 ± 0.009	0.16 ± 0.007	7.038	0.002**	0.019*	–	<0.0001**
Clauses per utterance	0.568 ± 0.005	0.572 ± 0.006	0.588 ± 0.004	0.783	0.461	–	0.049*	0.002**
Noun verb ratio	0.72 ± 0.017	0.69 ± 0.021	0.68 ± 0.015	0.649	0.526	–	–	–
Open–closed ratio	0.85 ± 0.014	0.82 ± 0.017	0.82 ± 0.012	0.424	0.656	–	–	–
Percentage of disfluencies	6.3 ± 0.52	6.2 ± 0.65	5.2 ± 0.47	1.496	0.231	–	–	–
Pause to word ratio	13.4 ± 0.77	11.3 ± 0.95	9.9 ± 0.68	4.033	0.022*	–	–	0.001**

Table displays estimates of means, covariates included in the model: age, sex and years of education.

*High D2R* schizophrenia patients with high dopamine D_2_ receptor medication, *low D2R* schizophrenia patients with low dopamine D_2_ receptor medication, *HC* healthy controls, *SE* standard error, *vs* versus, *MLU* mean length of utterance, *TTR* type-token ratio. For explanation of the language variables, see Table 1.

*Significant at the level of α = 0.05.

**Significant at the level of α = 0.01, *p*-values are Bonferroni corrected.

^a^Only significant *p*-values are reported.

### Relation with antipsychotic medication

A binary logistic regression model was used to investigate whether language variables could predict whether patients used a high or low D2R occupancy drug. Drug dosage was entered as a covariate. The optimal model had high predictive power (Nagelkerke approximation of *R*^2^ = 0.560), and the Hosmer-Lemeshow test for goodness-of-fit was non-significant (*p* = 0.932). The following language variables were included in the final model: mean pause duration, MLU, TTR, and speaking turn duration. Patients with high and low D2R occupancy could be classified with this model with a sensitivity of 80.0% and a specificity of 76.5%.

### Relation with antipsychotic medication and psychotic symptoms

To assess the effects of medication type and dose as well as psychotic symptoms, multivariate linear regression analyses were performed for each of the language variables. Psychotic symptoms, drug type (high or low D2R drugs) and drug dosage were entered as predictors. Unstandardized (*B*) and standardized (β) regression coefficients for each predictor in the separate models as well as the predictive utility of the entire model are reported in Table 4. The models for noun verb ratio and percentage of disfluencies were not significant. The variance in pause duration and the number of clauses per utterance were predicted solely by aspects of medication use (D2R occupancy for both, and dosage for pause duration). Speaking turn duration was only predicted by PANSS positive. Articulation rate, percentage of time speaking, MLU, TTR, and pause to word ratio were found to be affected by both symptoms and D2R occupancy.

**Table 4:** Regression analyses of predictors of language disturbances in schizophrenia patients.

Language variables	Significant predictor(s)	*B*	Confidence Interval B (95%)		β	Adjusted *R*^2^	*p*-value (uncorr.)	*p*-value (FDR corr.)
1. Articulation rate	PANSS positive	0.067	0.003	0.132	0.299	0.298	0.002**	0.004**
	PANSS negative	−0.069	−0.120	−0.017	−0.380			
	D2R occupancy	−0.359	−0.659	−0.059	−0.342			
2. Pause duration	D2R occupancy	0.089	0.010	0.167	0.380	0.114	0.049*	0.055
	Dosage (mg)^a^	0.0002	−0.00002	0.0005	0.304			
3. Speaking turn duration	PANSS positive	0.917	0.386	1.447	0.510	0.239	0.001**	0.002**
4. Percentage of time speaking	PANSS negative	−1.178	−1.664	−0.692	−0.574	0.539	<0.0001**	<0.0001**
	PANSS general	0.626	0.210	1.043	0.356			
	D2R occupancy	−6.525	−9.302	−3.748	−0.547			
5. MLU	PANSS negative	−0.498	−0.911	−0.085	−0.348	0.282	0.001**	0.003**
	D2R occupancy	−3.988	−6.384	−1.593	−0.480			
6. TTR	PANSS negative	0.003	0.0005	0.005	0.333	0.407	<0.0001**	<0.0001**
	PANSS general	−0.003	−0.004	−0.001	−0.412			
	D2R occupancy	0.025	0.013	0.037	0.558			
7. Clauses per utterance	D2R occupancy	−0.008	−0.017	−0.00001	−0.325	0.080	0.050	0.050
8. Open–closed ratio	PANSS negative	0.006	0.002	0.009	0.442	0.215	0.006**	0.009**
	D2R occupancy	0.022	−0.001	0.044	0.294			
9. Pause to word ratio	PANSS negative	0.261	0.042	0.481	0.360	0.206	0.008**	0.010**
	D2R occupancy	1.598	0.322	2.875	0.380			

The table displays unstandardized (*B*) and standardized (β) regression coefficient for each significant predictor, in a model for each of the language variables. The adjusted *R*^2^ and ANOVA *p*-values display the fit and significance of the full model. Predictors entered into the model were: PANSS positive, negative and general, D2R occupancy and chlorpromazine equivalent dose.

*PANSS* positive and negative syndrome scale, *D2R* dopamine D_2_ receptor, *FDR* false discovery rate, *uncorr.* uncorrected, *corr.* corrected, *MLU* mean length of utterance, *TTR* type-token ratio.

*indicates significance at the level of α = 0.05.

**indicates significance at the level of α = 0.01, No significant relations were found between PANSS and medication and noun verb ratio and percentage of disfluencies.

^a^Chlorpromazine equivalent dose.

## Discussion

Using computational language and speech analysis tools, this study evaluated the impact of antipsychotic medication type (high versus low D2R occupancy) on language disturbances in schizophrenia. We showed that patients who use high D2R occupancy drugs, such as aripiprazole, haloperidol, and risperidone, differ from patients who use low D2R occupancy drugs on total number of words and TTR, suggesting an effect of medication. Both patient groups differ from healthy controls on percentage of time speaking and clauses per utterance, suggesting illness effects. Overall, patients who use high D2R occupancy drugs have more severe negative language disturbances (i.e. slower articulation rate, increased and prolonged pauses and shorter utterances with fewer clauses), while less prominent disturbances are seen in patients who use low D2R occupancy drugs, such as clozapine and olanzapine. Language analyses were successful in predicting whether the recorded discourse belonged to a patient using high versus low D2R drugs. Finally, various language disturbances (MLU, TTR, pause to word ratio and clauses per utterance) were related to the use of high D2R occupancy drugs and the dosage of those drugs, rather than to the severity of the psychotic symptoms, which again suggests medication effects over illness effects.

As hypothesized, our results demonstrate that the use of high D2R occupancy drugs is associated with more severe language disturbances in schizophrenia compared to low D2R occupancy drugs, as reflected by reduced language production (i.e. total number of words produced) compared to low D2R occupancy drugs. Clinically, this might be described as alogia or poverty of speech, which is considered a negative symptom. This is most likely related to an increased hypodopaminergic state in the prefrontal cortex, as medication-induced decrease of dopamine in the prefrontal cortex impairs cognitive functioning in general and induces negative symptoms^34,35^.

Our results further demonstrated that patients who use high D2R occupancy drugs differ from healthy controls on several language parameters (pause duration, MLU, clauses per utterance, pause to word ratio), while low D2R occupancy patients do not or to a lesser degree. We interpret these results as indicative of two individual mechanisms of action. On the one hand, the finding that high D2R occupancy drugs are associated with increased pause rate, pause duration and reduced clauses per utterance, may be related to disturbances in language processing. Information transfer between prefrontal and temporal language-relevant regions is crucial for efficient language production^36,37^. A recent study by our group revealed that integrity of white-matter language pathways is associated with broad language disturbances in schizophrenia^38^. High D2R occupancy drugs may induce a hypodopaminergic stage and reduce information processing in language tracts, and thus give rise to language disturbances related to cognitive fluency or efficiency (i.e. pauses) and cognitive effort or complexity (i.e. MLU, clauses per utterance, TTR). Indeed, previous research in schizophrenia has shown that hypodopaminergic states are associated with white-matter integrity in the frontal cortex^39^. Moreover, dopamine replacement therapy in Parkinson’s disease is associated with both increased connectivity in white-matter language pathways improved and speech production^40^. On the other hand, patients using high D2R occupancy drugs spoke more slowly than controls (i.e. articulation rate), which can be related to blockage of the extrapyramidal system which has a slowing effect on articulation. In like manner, patients with Parkinson’s disease show a characteristic pattern of declining speech and articulation rate with illness progression^41–43^.

Our finding that high D2R occupancy drugs (such as risperidone) are associated with increased pausing, provides evidence that increased pausing is not related to sedative effects of antipsychotic drugs. Many of the low D2R occupancy drugs are highly sedating (e.g. olanzapine, quetiapine and clozapine), which is known to negatively influence cognitive performance^44^; instead we found increased pausing to be associated with less sedative antipsychotics.

A key question is whether the relative increase in language disturbances is caused by the use of high D2R occupancy drugs and should thus be regarded an adverse effect, or whether language disturbances in schizophrenia are relatively more severe in the high D2R occupancy group because they are better suppressed by low D2R occupancy drugs. The design of the current study does not allow for a discrimination between these mechanisms of action. Language disturbances are present in children who later develop psychosis^45^ and in youths at clinical high risk for psychosis^3,46,47^, in the absence of antipsychotic exposure, and are associated with the severity of psychotic symptoms in this group^48^. Moreover, language disturbances are also present in patients with bipolar disorder that do not use antipsychotic medication^49–51^. Within the small existing literature base dedicated to this topic, there is some evidence that language disturbances respond well to antipsychotic medication (haloperidol)^52^. Indeed, some have suggested that antipsychotic medication improves communication since it is associated with reduced incoherence and tangentiality^53^. However, there is also some evidence that antipsychotics reduce intelligibility of speech and induce poverty of speech^20,21^. Antipsychotics can also cause acute laryngeal dystonia^54^ or laryngeal dyskinesia^55^, causing stridor and thereby negatively impacting speech. Furthermore, in Huntington’s disease^56^, research shows that antipsychotic medication decreases speech rate and induces excessive loudness and pitch deviations. In contrast, dopamine replacement therapy in Parkinson’s disease has positive effects on speech tempo and prosody^57^. It is important to bear in mind that the field is in its early stages and our results are preliminary, and corresponding interpretations should, therefore, be regarded with caution. Moreover, not all language disturbances are the same; alogia, flat intonation (both negative symptoms), highly associated or incoherent language (both positive symptoms) might all be considered aberrant language production; however, the mental processes underlying these aberrations clearly differ. Therefore, the effects of antipsychotic medication on these language aberrations may differ as well. Replication in a larger sample is needed to fully understand the complex relation between language and antipsychotic medication.

The main limitations of this study include the absence of medication naïve or medication-free patients as well as a nonpsychotic patient group with antipsychotics, which precluded a pure assessment of the influence of antipsychotic medication on language production. Further, because a cross-sectional design was used, medication usage was not randomized and the language disturbances we observed could not be followed over time. Moreover, we could not rule out a bias in prescribing patterns of high and low D2R occupancy drugs, although clinical guidelines do not express any preference between antipsychotic drugs (except for clozapine). For this reason, a causal relation with the use of medication could not be established. Due to the design, we were unable to meaningfully examine the impact of neuropsychological deficits or other confounding variables (e.g., premorbid functioning, rapport, as well as illness-related factors such as sleep dysfunction, depression and paranoia) on language production. This remains an important topic for future research. It should be noted that we found an increased TTR in the patients as compared to the healthy controls. This is most likely an effect of sample size (total number of words produced), since the patients produced less words in total and TTR is known to be higher for smaller speech samples^58^. Furthermore, it is important to note that a large part of our high D2R occupancy patients used aripiprazole (65%). As stated above, aripiprazole is categorized as a strong D2R antagonist, although it also has some agonistic effects^59^. A related issue is that clozapine was grouped into the low D2R occupancy group, while clozapine is in general reserved for treatment of refractory patients with schizophrenia. Thus, replication in a large independent sample will be an important future research direction, in order to have sufficient statistical power to address the effects of each of the drugs individually. Of note, in the current study, we only evaluated the effects of dopaminergic receptor occupancy while most antipsychotic drugs also act on other neurotransmitter systems (e.g. serotonergic and anticholinergic receptors). For example, anticholinergic medication has been shown to negatively impact spoken language by inducing dryness of the oral and nasal mucosa^60^. Further research is needed to unravel the effects of these neurotransmitters on language disturbances. It should be noted that although no significant differences in language fluency were found between the groups, bilingualism is an important confounder in language research in schizophrenia. Ethnic minority groups in Western countries have an increased risk of developing schizophrenia, and more specifically linguistic distance to the majority language has been associated with increased psychosis risk^61^. Given the small sample size, this factor could not be fully explored in the current study, therefore further research is needed to assess the influence of bilingualism on language in schizophrenia.

We recognize and appreciate that there are several other approaches to quantify language disturbance in schizophrenia^33^. In the last decade, natural language processing analyses, specifically semantic space analyses and phonetic or prosodic methods, have been applied to language production in schizophrenia^62–66^. These are important developments that merit a future study designed to address the potential effects of medication on these specific analyses.

Our findings have several implications. First, as language is a highly important source of information in the psychiatric evaluative process, clinicians should be aware that poverty of speech in patients might be at least partly an effect of (highly dopaminergic) medication. Deteriorated language may, therefore, not necessarily be a sign of active psychosis. Second, since many schizophrenia patients require sustained pharmacological treatment to prevent relapses, research on language disturbances has been performed mostly in participants that are on antipsychotic medication. Further studies should acknowledge that the use of antipsychotic medication can influence their analyses.

In conclusion, we demonstrate that schizophrenia patients that use high D2R occupancy drugs (e.g. aripiprazole) have more severe language disturbances compared to patients that use low D2R occupancy drugs (e.g. olanzapine, quetiapine) and healthy controls, irrespective of the severity of their psychotic symptoms. Our results indicate that language disturbances are better treated by low D2R occupancy drugs, or that some language disturbances might (in part) be caused by dopaminergic effects of high D2R occupancy drugs. Language disturbances are common and greatly impact social and functional outcome and quality of life in schizophrenia. Further research is needed to evaluate possible iatrogenic effects of medication on spoken language.

## Methods

### Participants

A total of 81 participants, 41 patients with a schizophrenia spectrum disorder and 40 healthy controls were included at the University Medical Center Utrecht. Healthy controls were screened for previous or current mental illness using the Comprehensive Assessment of Symptoms and History (CASH)^67^ by a neuropsychologist. Patients were diagnosed by their treating psychiatrist; the diagnosis was confirmed using the outcome of the CASH or the Mini International Neuropsychiatric Interview 5.0.0. (M.I.N.I. Plus)^68^ by the first author or a neuropsychologist and a second rater for consensus diagnosis. Participants were included if they were (1) age eighteen or above and (2) a native speaker of Dutch. Bilingual participants were included if Dutch was (one of) their main language(s). An additional inclusion criterion for patients was the presence of a DSM-IV diagnosis of: 295.x (schizophrenia, schizophreniform disorder, schizoaffective disorder) or 298.9 (psychotic disorder NOS). General exclusion criteria were the presence of uncorrected hearing disabilities or speech impediments (such as stutter). Healthy controls were excluded in case of any current or previous mental illness, or a family history of psychotic symptoms. The severity of psychotic symptoms was assessed in all patients with the PANSS^69^. This study was reviewed and admitted by the Ethical Review Board of the University Medical Center Utrecht. Written informed consent was obtained from all participants. Participants received a small monetary award (ten euros).

### Language data acquisition and processing

To elicit spontaneous speech we (J.B., A.V. and trained research assistants) conducted semi-structured interviews varying from five to thirty minutes in length (average fourteen minutes). Participants were informed that the research involved the analysis of “general experiences”; only after completion of the interview were they told that the research also focuses on the way they speak. The interviews took place between December 2015 and March 2018.

A set of questions was used in the interview to control for potential variations in language due to the topic that was discussed. All questions concerned ‘neutral’ general life experiences; topics that could be expected to have markedly different emotional valence for patients and healthy controls were not addressed. For instance, topics such as “quality of life” or “health” were avoided. If for any reason a subject did not want to answer a question, the interviewer would move on to the next question. For a list of the questions, see supplementary Table 1.

An AKG-C544l head-worn cardioid microphone was used to record the subject’s speech. The first 39 interviews were conducted using a single AKG-C544l head-worn cardioid microphone, worn by the subject, recording both the interviewer’s and the subject’s speech onto a single channel. A second AKG-C544l head-worn cardioid microphone was used for the last interviews, resulting in a separate track for the subject and the interviewer. Speech was digitally recorded onto a Tascam DR40 solid-state recording device at a sampling rating of 44,100 kHz with 16-bit quantization.

The digitized recordings were analyzed using the Praat software^70^, which is standardly used for acoustic analyses of speech. Speakers’ speech signals were separated by hand onto two different tiers by J.B. and A.V. (i.e. two audio tracks were created, one for the participant and one for the interviewer). Each segment of speech was coded as belonging either to the participant or the interviewer. When both speakers spoke at the same time, that speech segment was coded as belonging to both speakers. The pause that arises with switching between speakers was attributed to the speaker following the pause. All speech segments of individual participants were recombined into new audio files, which each thus contained only the recording(s) of an individual participant’s speech, including pauses and other interruptions. Data files were blinded for diagnosis to prevent bias in separating the speaker. Inter-rater reliability for tier separation was assessed by having both raters perform tier separation for two of the files. Linguistic variables were then calculated for both audio files individually, after which intraclass-correlation-coefficients were calculated to assess the similarity in outcome measures for the different raters (which was 97.7 percent). All files were set to an average sound pressure level of 60 dB to avoid differences in the analyses based on speaking volume.

The ‘Praat Script Syllable Nuclei v2’^71^ was used to automatically obtain speech and articulation rates. The output of this script includes the following raw numbers: total number of syllables and total number of pauses. Pauses were defined as silences longer than 200 ms, since shorter silences in speech can still be related to the articulation of sounds such as plosives (e.g. the /p/, which introduces a short silence in the sound wave)^72^. The raw measures were calculated as a percentage of the duration of the participants’ audio track, since they are strongly dependent on the length of the interview. The participants’ audio file was transcribed using CLAN software according to the CHILDES manual^73^. In CLAN, the EVAL and FLUCALC functions were used to extract a collection of measures that reflect a person’s linguistic fluency and complexity, such as total number of words used, TTR, open–closed ratio (i.e., a ratio of content words versus function words) as well as pausing and disfluencies (see Fig. 1 and Table 1)^74^.

### Classification of antipsychotic medication

Patients were asked to bring a current list of the medication they used. The antipsychotic drugs were classified into different categories based on their mechanism of action. Drugs such as clozapine and quetiapine bind more loosely to the D2R than dopamine itself^28^. By contrast, typical antipsychotics such as haloperidol and risperidone are strong D2R antagonists since they bind more tightly to the receptor, which leads to higher receptor occupancy by the drug. Aripiprazole is also categorized as a strong D2R antagonist, although it also has some agonistic effects based on the cell type^59^. Patients were divided into two categories based on these different dopamine binding profiles, namely patients with (1) low D2R occupancy drugs (i.e. quetiapine, paliperidone, olanzapine and clozapine) or (2) high D2R occupancy drugs (i.e. aripiprazole, risperidone, flupentixol, amisulpride, and haloperidol)^75–77^. Antipsychotic drug dosages were recalculated into chlorpromazine equivalents to evaluate the effect of dosage between the drugs^78^.

### Data analysis

All analyses were performed in IBM SPSS Statistics version 25.0 for Windows. Participant characteristics were compared between groups using an analysis of variance (ANOVA) for continuous values, and a χ^2^ test for categorical values. To assess both the effect of antipsychotic medication and symptom severity, the following analyses were performed. (1) Between group (high D2R, low D2R and healthy controls) analysis of language features was obtained through a multivariate analysis of covariance (MANCOVA) by applying a general linear model (GLM). The MANCOVA assumptions of linearity, normality and homoscedasticity were checked visually by means of Q-Q plots and scatterplots of the residuals. P-values were Bonferroni corrected to control for Type 1 errors. (2) To investigate which language variables were associated with group membership (patients with low versus high D2R drugs), a backward binary logistic regression was performed. Predictors were the language variables, as well as age, gender and education level. (3) To model the effect of PANSS scores and the different types of antipsychotics (low versus high D2R drugs) and dosage on the measures of language, MRAs were performed. To account for possible biases due to multiple comparisons, false discovery rate (FDR) was employed^79^.

### Reporting summary

Further information on research design is available in the Nature Research Reporting Summary linked to this article.

## Supplementary information

Supplementary material

Reporting Summary

## Data Availability

The data that support the findings of this study are available on request from the corresponding author. The data are not publicly available due to them containing information that could compromise research participant privacy or consent.
